# Evaluation of skeletal age based on Greulich-Pyle method in Tehran

**DOI:** 10.1016/j.mex.2019.06.004

**Published:** 2019-06-25

**Authors:** Mohammadreza Ebrahimzade, Dariush Rahban, Aarman Baboli Bahmaei, Safdar Masoumi, Vahid Changizi, Mohammad Mirdoraghi

**Affiliations:** a Department of Anatomy, School of Medicine, Tehran University of Medical Sciences, Tehran, Iran; b Department of Nanomedicine, School of Advanced Medicine Technology, Tehran University of Medical Sciences, Iran; c Nursing and Emergency Department, Dezful University of Medical Sciences, Dezful, Iran; d Department of Epidemiology and Biostatistics, School of Public Health, Tehran University of Medical Sciences, Tehran, Iran; e Department of Radiology and Radiotherapy Technology, School of Allied Health Sciences, Tehran University of Medical Sciences, Tehran, Iran

**Keywords:** Determining of skeletal age based on Greulich-Pyle, Bone age, Chronological age, Greulich-Pyle (GP)

## Abstract

Although bone age plays a special role in determining the child’s age, there are some variations in skeletal growth of different people. The aim of this study was to compare the bone age with chronological age of children aged 2–18 years old in order to recognize whether Greulich-Pyle (GP) method could be reliable for Iranian children? The standard radiograph of Left hand was taken in 40 healthy subjects, then the bone age was determined according to GP. Mean ± SD bone ages were delayed 1.12 ± 0.65, 0.82 ± 1.34 and 0.10 ± 0.51 years than the mean chronological ages in 2.99–5.99, 10–13.99 and 14–17.99 age group, respectively; and advanced −0.33 ± 3.12 years in the 6–9.99 age group. In BMI levels <18.5, 18.5–24.9, 25–29.9 and ≥30, Mean ± SD bone ages in males were delayed 2.25 ± 0.21, 0.14 ± 0.55, 0.87 ± 0.41 and 4.05 ± 0.70 years than the mean chronological ages, respectively. In BMI range of 18.5–24.9 and BMI ≥ 30, Mean ± SD bone age in females was delayed 0.50 ± 0.49 and 0.45 ± 0.63 years than the mean chronological ages, respectively. For BMI < 18.5, Mean ± SD bone age in females were advanced −0.40 ± 2.69 years than mean chronological ages. Considering these differences, Iranian boys may have a different pattern of bone growth from GP standards.

## Graphical abstract

**Figure:**
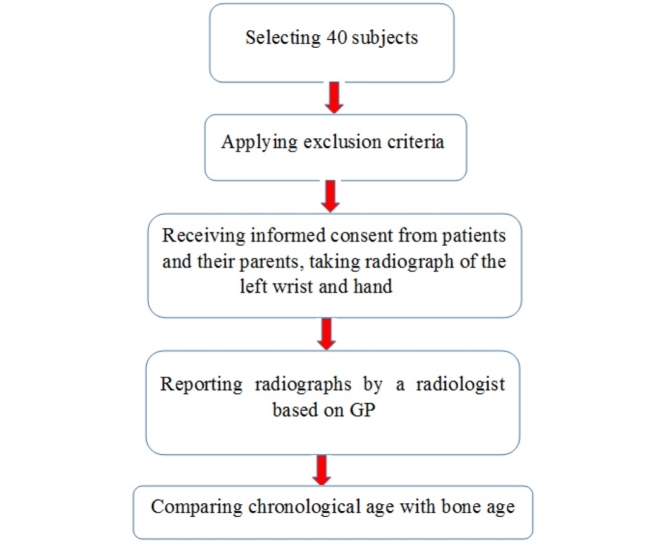

**Specifications Table**

**Table:** 

Subject area:	Medical physics.
More specific subject area:	Evaluation of skeletal age based on Greulich-Pyle method.
Type of data:	Graph, table.
Protocol name:	Determining of skeletal age based on Greulich-Pyle.
Experimental design:	To compare bone age with chronological age, 40 children were randomly selected. After receiving informed consent from patients and their parents, radiograph of the left wrist and hand was taken. Based on GP, all radiographs were reported by a radiologist who was of the patient’s chronological age [[1], [2], [3], [4]].

**Value of the Protocol**

**Table:** 

Determining of skeletal age based on Greulich-Pyle method, recognizing the differences in skeletal growth of Iranian children compared with GP method.

## Protocol data

• The data demonstrate that variations in skeletal growth in children aged 6–9.99 and 10–13.99 years old are greater than those evaluated by the Greulich and Pyle atlas, and that there are significant differences between bone and chronological ages.
• The categorizing groups based on BMD shows a great differences among our Mean ± SD chronological and bone ages in those male subjects who are underweight (BMD < 18.5) and obese (BMI ≥ 30). The Mean ± SD of difference of bone and chronological ages in male normal children (18.5 ≤ BMI ≤ 24.9) and overweight children (25 ≤ BMI ≤ 29.9) were 0.14 ± 0.55 and 0.87 ± 0.41 years, respectively.
• The Mean ± SD difference of chronological and bone ages in female subjects that are underweight, normal, and obese is −0.40 ± 2.69, 0.50 ± 0.49 and 0.45 ± 0.63, respectively.
• The variation in skeletal growth of Iranian children and children’s group that the GP Atlas was taken could be related to environmental, economic, social, cultural and racial differences. Although this discrepancy is greater in boys than girls, so it may be affected by gender in Iran. Thus, GP Atlas could be more accurate to determine bone age in Iranian girls than boys.

## Description of protocol

**Fig. 1:**
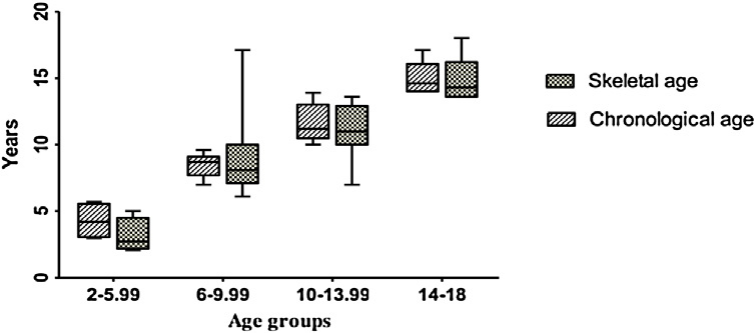
The distribution of chronological ages and skeletal ages according to the Greulich-Pyle atlas for each age group.

The Mean ± SD bone and chronological ages, weight, height and BMI of subjects based on gender are presented. Although the Mean ± SD BMI in boys was more than girls, the difference between Mean ± SD chronological and bone age in boys was lower than that of girls. The difference of Mean ± SD of chronological and bone ages in group 1 was more than other aged grouped (Table 1, Fig. 1). Also, the highest Mean ± SD BMI was observed in group 3 (Table 2). The number of male subjects and Mean ± SD chronological and bone ages according to BMI ranges are shown in Table 3. In the BMI < 18.5, 18.5 ≤ BMI ≤ 24.9, 25 ≤ BMI ≤ 29.9 and BMI ≥ 30, Mean ± SD bone ages in boys were −0.40 ± 2.69, 0.50 ± 0.49, 0.45 ± 0.63, 4.05 ± 0.70 years delayed than the Mean ± SD chronological ages, respectively. Also, Mean ± SD chronological and bone ages in girls according to BMI ranges are shown in Table 4. In the BMI < 18.5, 18.5 ≤ BMI ≤ 24.9 and BMI ≥ 30 groups of girls, Mean ± SD bone ages were –0.40 ± 2.69, 0.50 ± 0.49, 0.45 ± 0.63 years delayed than the Mean ± SD chronological ages, respectively. In the 10–13.99 years age group, mean bone age was delayed 1.34 ± 0.31 years than mean chronological age, and it was statistically significant (Table 5) (P = 0.018).

**Table 1:** Mean ± SD age and anthropometric characteristics of 40 healthy children.

	Girls	Boys
*n*	24	15
Bone age (y)	10.59 ± 3.22	9.16 ± 4.46
Chronological age	10.55 ± 2.73	10.30 ± 3.93
Weight (kg)	36.87 ± 11.96	38.93 ± 16.27
Height (cm)	1.37 ± 23.03	1.27 ± 34.82
BMI (kg/m^2^)	20.28 ± 8.37	23.64 ± 5.29

**Table 2:** Mean ± SD chronological age, bone age, difference between chronological age and bone age, and BMI in healthy individuals of aged grouped.

Age groups	*n*	Chron. age (y)	Bone age (y)	Df of both age	BMI
2.99–5.99	4	4.27 ± 1.32	3.15 ± 1.28	1.12 ± 0.65	20.46 ± 7.31
6–9.99	11	8.46 ± 0.81	8.80 ± 3.03	−0.33 ± 3.12	21.24 ± 11.46
10–13.99	18	11.53 ± 1.30	10.71 ± 2.00	0.82 ± 1.34	22.16 ± 5.83
14–18	6	15 ± 1.24	14.90 ± 1.74	0.10 ± 0.51	21.15 ± 2.89

**Table 3:** Mean ± SD chronological age, bone age, weight, and height in healthy boys of Aged grouped.

BMI groups	*n*	Chron. age (y)	Bone age (y)	Mean ± SD difference (y)
<18.5	2	7.75 ± 3.74	5.50 ± 3.53	2.25 ± 0.21
18.5–24.9	7	12.08 ± 2.88	11.94 ± 3.27	0.14 ± 0.55
25–29.9	4	8.07 ± 5.68	7.20 ± 5.67	0.87 ± 0.41
≥30	2	11.05 ± 0.070	7.00 ± 0.00	4.05 ± 0.70
Total	15	10.30 ± 3.93	9.16 ± 4.46	1.14 ± 1.44

**Table 4:** Mean ± SD chronological age, bone age, weight, and height in healthy girls of Aged grouped.

BMI groups	*n*	Chron. age (y)	Bone age (y)	Mean ± SD difference (y)
<18.5	14	10.10 ± 2.50	10.50 ± 3.30	−0.40 ± 2.69
18.5–24.9	8	11.87 ± 2.94	11.37 ± 3.18	0.50 ± 0.49
≥30	2	8.50 ± 2.12	8.05 ± 2.75	0.45 ± 0.63
Total	24	10.55 ± 2.73	10.59 ± 3.22	−0.03 ± 2.09

**Table 5:** Differences between bone age and chronological age in healthy Iranian children, grouped by age grouped.

Age	n	Chronological ages (years˜SD)	Skeletal ages (years˜SD)	Differences (years˜SD)	BMI	T	P-value	df
2–5.99	4	1.32 ± 0.66	3.15 ± 2.56	0.65 ± 0.32	20.46 ± 7.31	3.43	0.41	3
6–9.99	11	0.81 ± 0.24	8.80 ± 10.01	3.12 ± 0.94	21.24 ± 11.46	−0.35	0.072	10
10–13.99	18	1.30 ± 0.30	10.71 ± 8.46	1.34 ± 0.31	22.16 ± 5.83	2.60	0.018	17
14–18	6	1.24 ± 0.50	14.90 ± 4.26	0.51 ± 0.21	21.15 ± 2.89	0.47	0.65	5

## Materials and methods

The aim of this methodology is to determine the differences between bone age and chronological age in Iranian children which could lead more accurate estimation of bone age. To compare bone age with chronological age, 40 children were randomly selected. Subjects aged between 2 and 18 years living in Tehran City, Iran. Exclusion criteria were history of systemic diseases more than 1 month, history of chronic systemic diseases, history of hospitalization for more than a week, and people with height or weight above the percentile of 97% or below 3 percent percentile. After receiving informed consent from patients and their parents, radiograph of the left wrist and hand was taken. Based on GP [5], all radiographs were reported by a radiologist who was of the patient’s chronological age. These individuals were divided into four groups: 2–5.99, 6–9.99, 10–13.99 and 14–18 years old.

### Statistical analysis

The normality of data was checked out by Shapiro–Wilk Test. For each group, mean ± SD of bone age, chronological age, weight, height and BMI (Body Mass Index) were determined; then data were analyzed using One-Sample *T*-Test by SPSS.

## Conclusion

Although GP method is reliable and known method, there are some variations in skeletal growth of the Iranian children and children’s group that the GP Atlas was taken that could be related to environmental, economic, social, cultural and racial differences. Since this discrepancy is greater in boys than girls, it may be affected by gender in Iran. Therefore, GP Atlas could be more accurate to determine bone age in Iranian girls than boys.
